# Single circulating tumor cell sequencing for monitoring

**DOI:** 10.18632/oncotarget.1026

**Published:** 2013-05-10

**Authors:** Martina Auer, Ellen Heitzer, Peter Ulz, Jochen B Geigl, Michael R Speicher

**Affiliations:** Institute of Human Genetics, Medical University of Graz, Harrachgasse, Austria; Institute of Human Genetics, Medical University of Graz, Harrachgasse, Austria; Institute of Human Genetics, Medical University of Graz, Harrachgasse, Austria; Institute of Human Genetics, Medical University of Graz, Harrachgasse, Austria; Institute of Human Genetics, Medical University of Graz, Harrachgasse, Austria

To date, comprehensive sequencing efforts have elucidated the genomic landscapes of multiple cancer entities and increased our understanding of tumor biology [1]. The identification of tumor-specific somatic mutations associated with respective cellular pathways has paved the way for targeted therapies [2]. Hence somatic mutations may serve as predictive biomarkers of tumor response and may guide clinicians in their decisions about treatment options. The inclusion of genomic data in treatment decisions has frequently been referred to as “personalized medicine” or “precision medicine”. Although personalized medicine appears to be extremely attractive, a potential drawback is that tumor genomes are instable and that clones resistant to a given therapy can take over. As a consequence sequencing information obtained from primary tumor genomes may be of limited value if, for example, a relapse occurs a long time after diagnosis.

Hence, we and others pursued ways to extract information about tumor genomes at various time points during a disease course by non-invasive means. To this end a cancer patient's blood represents a “liquid biopsy”, as it may contain both circulating tumor cells (CTCs) and DNA (ctDNA). CTCs are very rare cells (estimated frequency: 1 CTC per 1×10^9^ normal blood cells) found in the blood of most patients with solid tumors [3]. The most widespread CTC detector is the CellSearch system, which traps CTCs using an antibody against epithelial cell adhesion molecule (EpCAM) that is found on the tumor cells but not on blood cells. Subsequent identification of CTCs is based on cytokeratin (CK)-positivity and negativity for the leukocyte common antigen CD45. The CellSearch system has Food and Drug Administration approval for monitoring patients with metastatic breast, prostate, and colorectal cancer [3].

**Figure 1:**
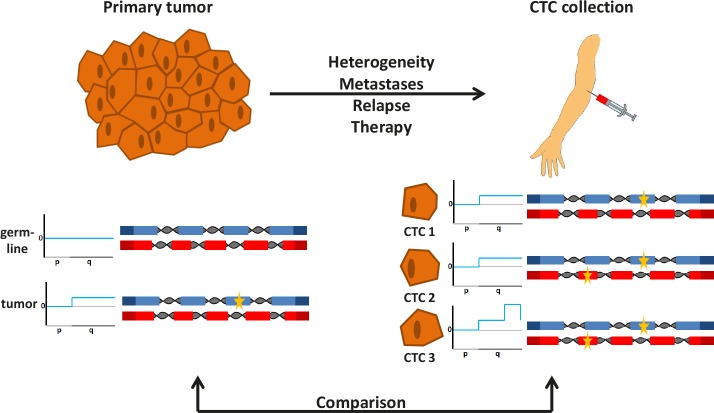
Analyses of the genomes of a primary tumor and CTCs The genomes of tumor cells from a primary tumor and nonmalignant cells from the same patient are analyzed for copy number changes and mutations at nucleotide level (shown on the left side). The comparison of variants in nonmalignant cells (i.e. the germline) with those observed in tumor cells allows the detection of tumor-specific, somatic changes. Here, no copy number variations were observed in the germline (illustrated exemplarily for the short (p) and long (q) arm of one chromosome, and no mutations were identified in two genes (shown with blue and red exons, respectively). In contrast, tumor cells had a copy number change (indicated by a gain of the long arm) and a mutation (indicated by the yellow star in the blue gene). While analysis of the primary tumor is usually done at the time of diagnosis, CTCs can be analyzed at any time during the disease course and are obtained simply by blood collection (right side). The genomes of the CTCs may differ from the initial analysis of the primary tumor for various reasons (see text). If CTCs are analyzed on a cell-by-cell basis, they may reveal heterogeneity. For example, CTC1 has the same changes as the primary tumor, CTC2 has an additional mutation in another gene (indicated by a yellow star in the red gene), whereas CTC3 has in addition a novel copy number change (i.e. high level amplification on the q-arm). Comparison between the genome of the primary tumor with the genomes from CTCs provides information about the evolution of the tumor genome and potentially novel biomarkers.

In addition to the surge of published whole tumor genome sequencing data, significant progress was made in single cell genome analyses technologies [4]. The combination of efficient CTC capturing devices with novel single cell sequencing approaches should significantly advance the area of CTC-based liquid biopsies (Figure 1). Indeed, we recently succeeded in analyzing genomes of CTCs from patients with colorectal cancer (CRC), which were captured using the CellSearch system. After whole-genome amplification (WGA) we employed massive parallel sequencing of a panel of 68 CRC-associated genes [5]. In addition, we applied array-CGH to establish copy number profiles of the same CTCs. The results were compared to the mutation spectrum observed in the primary tumors of the same patients (as outlined in Figure 1). To the best of our knowledge, our study [5] represents the most detailed analysis of CTC genomes published to date.

Mutations in known driver genes (e.g., *APC*, *KRAS*, or *PIK3CA*), which we detected in the primary tumor, were also detected in corresponding CTCs. However, we also observed mutations exclusively in single CTCs and referred to those as “private CTC mutations”. The occurrence of such private mutations allows several interpretations, including that they may represent just WGA/sequencing artifacts. Other studies using next-generation sequencing of single cancer cell WGA products addressed WGA and sequencing errors by combining data of several cells to deliver reliable nucleotide variant calls. However, such a strategy is not applicable to CTCs because of their rarity and the limited number of available CTCs for analysis.

To address this issue we developed a different approach where we performed additional ultra-deep sequencing of the primary tumor and applied a customized statistical algorithm for analysis. This analysis revealed that indeed most mutations initially found only in single CTCs were also present at subclonal level in the primary tumors from the same patient. This suggests that single CTC analyses may unravel the heterogeneity of primary tumors with high resolution.

We also found evidence that CTC genome analysis may identify novel changes of clinical relevance. For example, in one patient we observed *CDK8* amplification on chromosome 13q12.13 in 9 of 10 CTCs that was not present in the parts of the primary tumor we analyzed. This amplification may represent a viable target for CDK inhibitors, which are currently in clinical trials.

At present, the most cost- and time-efficient CTC sequencing strategy is still an open question. With our growing knowledge of cancer genomes [1] it may be sufficient to focus sequencing efforts on alterations which promote tumorigenesis and their associated signaling pathways. Thus, for extracting prognostic and predictive information from CTC genomes focused sequencing approaches after targeted enrichment instead of whole CTC sequencing may have advantages for clinical purposes. In addition, we and others [6-8] demonstrated that characteristics of the tumor genome can be deduced by whole plasma DNA sequencing. Indeed, the ctDNA within plasma appears to reflect characteristics of the current status of the tumor genome at the time of blood collection. Hence in addition to CTCs detailed plasma DNA analyses appear to evolve to an additional tool for the reconstruction of complex tumor genomes from the peripheral blood, which is a step forward to realize personalized medicine efforts.
